# CEST MRI reveals nicotine-induced alterations in glutamate-associated molecular connectivity in the mouse brain

**DOI:** 10.3389/fnins.2026.1815092

**Published:** 2026-06-16

**Authors:** Joel Jarusek, Mariano G. Uberti, Adarsha Bhattarai, Dongming Peng, Aditya N. Bade, Yutong Liu

**Affiliations:** 1 Department of Radiology, University of Nebraska Medical Center, Omaha, NE, United States; 2 Department of Physics, University of Nebraska at Omaha, Omaha, NE, United States; 3 Department of Electrical and Computer Engineering, University of Nebraska-Lincoln, Omaha, NE, United States; 4 Department of Pharmacology and Experimental Neuroscience, University of Nebraska Medical Center, Omaha, NE, United States

**Keywords:** CEST MRI, glutamate, graph theory, molecular connectivity, mouse brain networks, neuroimaging, nicotine

## Abstract

**Introduction:**

Understanding how neurotransmitter systems organize into large-scale networks is essential for elucidating the mechanisms through which drugs, diseases, and behavioral states alter brain function. Existing imaging modalities such as functional MRI (fMRI) and positron emission tomography (PET) provide measures of hemodynamic and metabolic connectivity, but cannot noninvasively map neurotransmitter-associated networks with high spatial resolution. Herein, we introduce a chemical exchange saturation transfer (CEST) MRI-based framework for mapping glutamate-associated molecular connectivity and apply it to characterize nicotine-induced network reorganization in the mouse brain.

**Methods:**

Male C57BL/6 mice underwent dynamic glutamate-weighted CEST (gluCEST) MRI before and after seven days of nicotine exposure. Regional glutamate-weighted CEST time series were extracted from 51 brain regions, and connectivity was evaluated using within-subject temporal correlation and inter-subject covariance analyses.

**Results:**

Graph theory analyses identified four baseline glutamate-associated modules involving frontal-sensorimotor, cortico-hippocampal, intra-hippocampal, and cortico-striatal circuits. Nicotine exposure attenuated these baseline networks in analyses performed without global signal regression (GSR) and with conditional GSR, whereas full GSR reduced the apparent magnitude of these effects. Nicotine also reduced nodal strength in the CA1 and insular cortex. In contrast, nicotine selectively strengthened a thalamo-striato-motor circuit involving the motor cortex, mediodorsal and ventral thalamic nuclei, and caudoputamen. This pattern remained evident even under full GSR. Subject-level covariance analysis confirmed widespread nicotine-induced attenuation of glutamate-associated connectivity and revealed a distinct sensory-limbic module involving the lateral geniculate nucleus, amygdala, and piriform cortex that was selectively disrupted following nicotine exposure.

**Discussion:**

These results demonstrate the feasibility of dynamic gluCEST MRI for mapping glutamate-associated molecular connectivity *in vivo* and detecting pharmacologically induced network remodeling. This approach provides a noninvasive platform for investigating glutamatergic dysregulation in addiction, neuropsychiatric disorders, and therapeutic response.

## 1 Introduction

Brain connectivity is commonly studied using diffusion MRI, functional MRI (fMRI), and positron emission tomography (PET) (Sporns et al., 2005; Bullmore and Sporns, 2009; Sporns, 2013; Sporns, 2016; Hagmann et al., 2010). Diffusion MRI maps structural pathways. Functional MRI provides hemodynamic-based functional connectivity (Buxton, 2012), and PET enables molecular network mapping (Yakushev et al., 2017; Sala and Perani, 2019; Carli et al., 2021; Severino et al., 2025; Hahn et al., 2024; Jamadar et al., 2021; Amend et al., 2019; Hahn et al., 2025). However, neither modality can noninvasively map metabolic or neurotransmitter-associated connectivity with high spatial resolution without substantial limitations. Functional MRI relies on indirect blood oxygenation level dependent (BOLD) contrast, and PET requires radiotracer administration and has limited spatial resolution.

Chemical exchange saturation transfer (CEST) MRI provides metabolite-weighted contrast with substantially higher spatial resolution and without exogenous tracers (Pankowska et al., 2019; van Zijl and Yadav, 2011; Vinogradov et al., 1997; Ward et al., 2000; Sajja et al., 2016). Among CEST approaches, glutamate-weighted CEST (gluCEST) MRI enables sensitive detection of glutamate-associated contrast (Bagga et al., 2018; Cai et al., 2012; Debnath et al., 2020; Hadar et al., 2021; Jia et al., 2020; Li et al., 2020; Neal et al., 2019) and therefore offers a potential framework for mapping glutamate-associated molecular connectivity. Because glutamate is the principal excitatory neurotransmitter in the central nervous system (CNS) and plays a critical role in synaptic transmission, neuroplasticity, and addiction-related circuitry, mapping glutamate-associated connectivity may provide a unique perspective on large-scale brain network organization.

Technical considerations regarding global signal fluctuations were also incorporated into the analysis. In conventional BOLD fMRI, global signal regression (GSR) is sometimes applied to reduce the global signal, which reflects a mixture of motion-, respiration-, and physiology-related variation, although the global signal may also contain biologically meaningful neural information (Grandjean et al., 2023; Murphy and Fox, 2017; Li et al., 2019; Saad et al., 2012). While GSR can effectively suppress widespread non-neuronal artifacts, it may also remove meaningful neural information and introduce spurious negative correlations. Thus, the use of GSR remains one of the most debated preprocessing choices in functional connectivity research (Saad et al., 2012; Liu et al., 2017; Murphy et al., 2009). Because the characteristics of global signal variation in dynamic gluCEST MRI remain incompletely understood and may differ from BOLD fMRI, we implemented complementary preprocessing pipelines with and without GSR. This approach allowed us to assess the robustness of nicotine-related glutamate-associated connectivity changes across preprocessing strategies and distinguish stable network findings from those influenced by global signal handling.

Nicotine was selected as a pharmacologic perturbation because glutamatergic signaling is critically involved in nicotine reinforcement, neuroadaptation, and addiction-related circuit remodeling. Male C57BL/6 mice were used because this strain is widely employed in nicotine-related preclinical studies and provides a well-characterized background for evaluating nicotine-induced neurobiological responses (Matta et al., 2007; Alasmari et al., 2019; Bade et al., 2017; Cohen and George, 2013).

Overall, in this study, we introduce a CEST MRI-based framework for mapping glutamate-associated molecular connectivity and apply it to evaluate nicotine-induced alterations in the mouse brain. Moreover, the techniques developed herein can be readily extended to other biomolecules, including glucose, creatine, and myo-inositol. By enabling direct assessment of metabolite-associated connectivity with spatial resolution comparable to structural MRI, this approach provides a powerful new tool for probing molecular network organization and its alterations in health, disease, and response to therapeutic interventions.

## 2 Materials and methods

### 2.1 Mice and nicotine administration

Male C57BL/6 mice (*n* = 8, age = 18–20 weeks) received daily intraperitoneal (IP) injections of nicotine (10 mg/kg/day) for seven consecutive days. C57BL/6 mice were selected because this strain is widely used in nicotine and addiction-related preclinical studies and provides a well-characterized background for evaluating nicotine-induced neurobiological responses (Matta et al., 2007; Alasmari et al., 2019; Bade et al., 2017; Cohen and George, 2013). The selected dose was based on prior preclinical studies investigating long-term nicotine exposure and nicotine-induced neurobiological modulation in rodents (Matta et al., 2007; Alasmari et al., 2019; Bade et al., 2017; Cohen and George, 2013). Rodents metabolize nicotine substantially faster than humans, with shorter nicotine and cotinine half-lives; therefore, relatively higher systemic doses are commonly used in rodent studies to achieve sustained brain nicotine exposure and neurochemical effects comparable to chronic human nicotine exposure. The nicotine solution was prepared by dissolving nicotine bitartrate (Sigma-Aldrich, St. Louis, MO) in PBS. All animal procedures were conducted in accordance with the Association for Assessment and Accreditation of Laboratory Animal Care guidelines and approved by the Institutional Animal Care and Use Committee (IACUC) of the University of Nebraska Medical Center (UNMC). All UNMC animal ethical standards in compliance with the National Institutes of Health were followed. Throughout the experimental period and during MRI preparation, mice did not exhibit overt signs of toxicity, excessive sedation, or respiratory distress, and physiologic monitoring remained within expected ranges.

### 2.2 MRI acquisition

MRI experiments were performed on a 7 Tesla system (Bruker PharmaScan, Bruker BioSpin, Billerica, MA) operating with ParaVision 7 using a Bruker-built mouse brain volume coil. Each mouse underwent two MRI sessions: a baseline scan performed prior to the first nicotine administration, followed immediately by the initial nicotine injection, and a second scan acquired one hour after the final nicotine injection on day 7. During imaging, anesthesia was maintained with isoflurane delivered in oxygen at 1 L/min, with the isoflurane level adjusted to maintain a respiratory rate between 40 and 80 breaths per minute. Isoflurane was selected based on recommendations from a recent multi-center consensus analysis of rodent functional connectivity imaging (Grandjean et al., 2023). Body temperature was maintained above 35 °C using a water-heated animal bed integrated into the Bruker animal table and continuously monitored with a rectal temperature probe (Small Animal Instrumentation, Inc., Stony Brook, NY, USA). Respiratory rate was monitored throughout the imaging session.

An imaging session included localized shimming within an ellipsoidal volume positioned over the mouse brain using Bruker MAPSHIM method, a complete CEST acquisition with 51 frequency offsets ranging from −5 to 5 ppm in 0.2 ppm increments. Dynamic gluCEST MRI was performed using a single-frequency approach targeting glutamate-associated contrast at 3.0 ppm. Dynamic CEST datasets were acquired at apparent frequency offsets of 2.9 and 3.1 ppm in an alternating sequence (2.9 ppm followed by 3.1 ppm), repeated 100 times, resulting in 100 dynamic datasets acquired at each offset. The actual frequency offset of each voxel was then determined using the WASSR-based B0 map generated using the complete CEST dataset (Kim et al., 2009). The glutamate-weighted signal at 3.0 ppm was generated by interpolation from the signals at the two corrected neighboring offsets. In addition, mirrored datasets were acquired at −2.9 and −3.1 ppm, corrected using the B0 map, and interpolated to generate the reference dataset at −3.0 ppm. This reference dataset was used for signal normalization and motion correction. Dynamic gluCEST acquisition parameters were: RF saturation power = 2 μT with saturation duration = 1 s; 15 slices covering the whole brain (slice thickness = 0.5 mm with 0.5 mm gap); field of view = 20 × 20 mm^2^; acquisition matrix = 96 × 64; and 100 temporal datasets acquired per offset. The temporal resolution of the reconstructed dynamic gluCEST time series was 35 s per time point. The total scan time of an imaging session was less than 80 min.

### 2.3 Dynamic gluCEST data preprocessing

After interpolation of the dynamic gluCEST datasets at 3.0 ppm and generation of the reference datasets at −3.0 ppm as described above, motion correction was performed by registering each interpolated dynamic gluCEST dataset at 3.0 ppm to the interpolated reference dataset at −3.0 ppm. The motion-corrected dynamic gluCEST datasets were then normalized using the corresponding −3.0 ppm reference image.

To evaluate the effect of global signal regression (GSR) on connectivity mapping, three preprocessing strategies were tested: (1) without GSR, (2) conditional GSR, in which GSR was applied only when the global negative index (GNI) described by Chen et al. (2012) was < 3, and (3) full GSR applied to all datasets. The GNI was used as a heuristic indicator of the extent to which GSR may introduce widespread negative correlations into the connectivity structure. Lower GNI values indicate datasets in which the impact of GSR-induced anti-correlations is expected to be smaller. The threshold value of GNI < 3 was adopted as a pragmatic preprocessing sensitivity criterion based on the framework proposed by Chen et al. (2012), rather than as a universally established biological cutoff. The purpose of this conditional approach was to reduce the potential introduction of widespread artificial anti-correlations while still allowing evaluation of the effects of GSR on glutamate-associated connectivity patterns.

The normalized dynamic gluCEST datasets were subsequently registered to an MRI-based mouse brain atlas developed by the Australian Mouse Brain Mapping Consortium (AMBMC) (Ullmann et al., 2014) for spatial normalization and glutamate-associated network analysis using graph theory methods.

Because dynamic gluCEST imaging requires repeated acquisitions over an extended imaging period, the resulting time series may be susceptible to signal fluctuations related to noise, physiological variability, scanner drift, and motion-related effects. However, no substantial temporal drift was observed in the current datasets. Data preprocessing was carried out using customized MATLAB scripts developed in-house.

### 2.4 Brain parcellation and computation of glutamate-associated connectivity

A total of 51 brain regions of interest (ROIs) were defined listed in Glossary based on AMBMC mouse brain atlas, consisting of 25 anatomically paired left/right brain regions and one midline region spanning both hemispheres. Left and right hemispheric regions were treated as independent ROIs in all analyses. These ROIs covered major structures across the cortex, hippocampus, basal ganglia, diencephalon, and cerebellum. Voxel intensities within each ROI were averaged at every time point to generate the region-specific gluCEST time series. Glutamate-associated connectivity was estimated by computing the Pearson correlation coefficient between all pairs of regional time series, resulting in a 51 × 51 symmetric correlation matrix. The main diagonal, representing self-correlations, was excluded from further analysis. All off-diagonal correlation coefficients were then Fisher r-to-z transformed to improve normality and enable statistical analysis (Christiaen et al., 2020).

### 2.5 Computation of network metrics and statistical analysis

Graph theory connectivity metrics were calculated using the Brain Connectivity Toolbox (BCT) (Rubinov and Sporns, 2010). To ensure equal network density across subjects and conditions, correlation matrices were proportionally thresholded to retain the strongest 5% of edges, consistent with previous work (Christiaen et al., 2020). Statistical comparisons of connectivity metrices between the pre-nicotine condition (before nicotine administration) and the post-nicotine condition (after nicotine administration) were conducted using paired t-tests in GraphPad Prism. Multiple comparisons were controlled using the false discovery rate (FDR) method, and corrected *p* values < 0.05 were considered statistically significant.

### 2.6 Subject-level (inter-subject) connectivity analysis

In addition to estimating connectivity using within-subject temporal correlations of gluCEST signals, we also evaluated connectivity across subjects using a subject-level covariance approach. For this method, the gluCEST time series for each subject were averaged to obtain one mean value per region per subject. These regional mean values were then treated as a subject series, and connectivity was quantified by calculating the Pearson correlation coefficient between regional values across subjects. Whereas the temporal correlation method measures the synchrony of glutamate-weighted signal fluctuations over time within individual subjects, the subject-level correlation method captures inter-subject covariation in glutamate levels across brain regions.

## 3 Results

### 3.1 Connectivity specificity using seed-based correlation analysis

**Figure 1:**
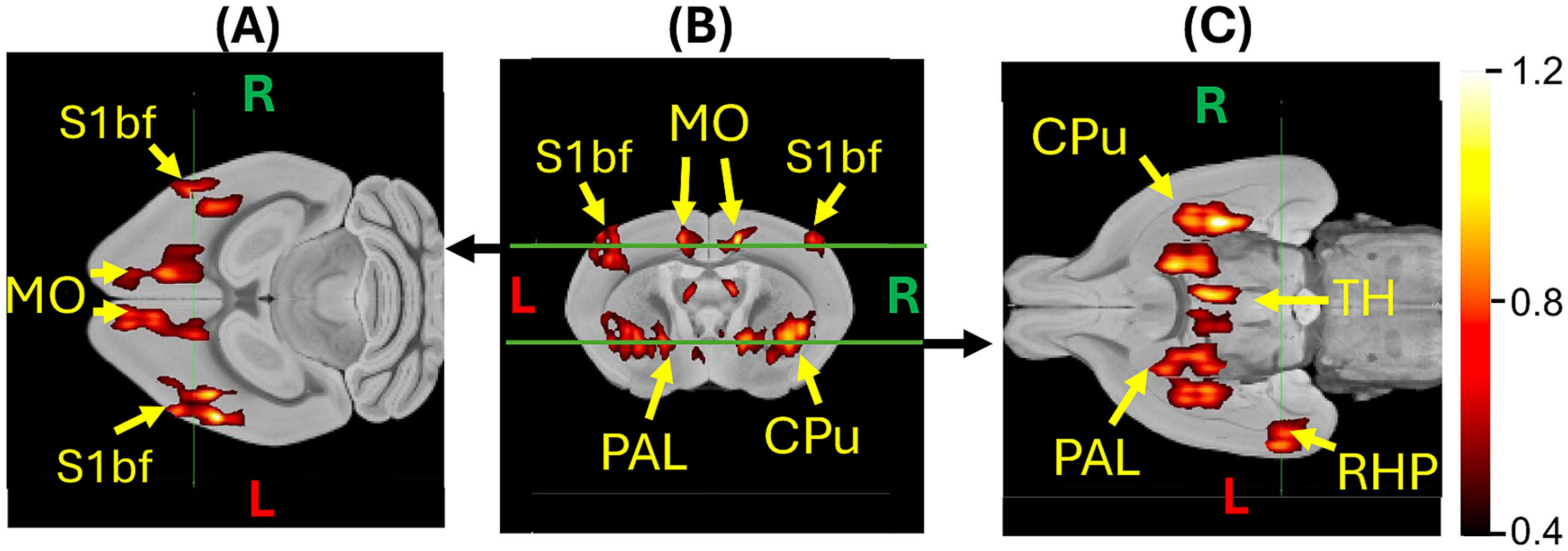
Quality assessment and connectivity validation of dynamic gluCEST data. **(A-C)** Seed-based correlation analysis using the right primary somatosensory barrel field (S1bf) as the seed region. Voxel-wise correlation maps were Fisher Z-transformed, and regions with Z > 0.4 are displayed. The right S1bf showed strong correlations with the contralateral S1bf, motor cortex (M), pallidum (PAL), caudatoputamen (CPu), and thalamus (TH) bilaterally, as well as with the left retrosplenial hippocampus (RHP).

Representative gluCEST maps from several middle brain slices acquired before and after nicotine exposure were shown in Supplementary Figure 1. Dynamic gluCEST data demonstrated robust quality and reliable connectivity patterns. This analysis was intended primarily as a qualitative validation of anatomically plausible glutamate-associated connectivity patterns rather than as a primary inferential endpoint. To examine data quality, we evaluated the specificity of connectivity in the right primary somatosensory (S1) barrel field (S1bf) by performing seed-based correlation analysis for each mouse. The right S1bf was selected because it is one of the most consistently characterized and reproducible functional networks in rodent brain connectivity studies. Prior rodent resting-state fMRI studies and recent multi-center consensus analyses have demonstrated robust bilateral homotopic S1bf connectivity across animals and preprocessing pipelines (Grandjean et al., 2023), making this region a commonly used reference for evaluating biologically plausible connectivity structure and data quality. Correlation coefficients between the right S1bf and all brain voxels were Fisher Z-transformed. For visualization purposes, regions exceeding a threshold of Z > 0.4 are shown in Figures 1A–C. Strong bilateral connectivity was observed between the right and contralateral S1bf, as well as with the motor cortex (M), pallidum (PAL), caudatoputamen (CPu), and thalamus (TH). In addition, significant connectivity was also detected with the left retrosplenial hippocampus (RHP), highlighting extended connections beyond the sensorimotor network. The seed-based correlation analysis demonstrated that the dynamic gluCEST data exhibited high quality and strong connectivity specificity. The right S1bf showed robust, anatomically appropriate correlations with contralateral S1bf, motor cortex, subcortical sensorimotor structures, and retrosplenial hippocampus, consistent with known glutamatergic pathways.

### 3.2 Nicotine-induced reorganization of glutamate-associated networks

#### 3.2.1 Data processed without GSR

**Figure 2:**
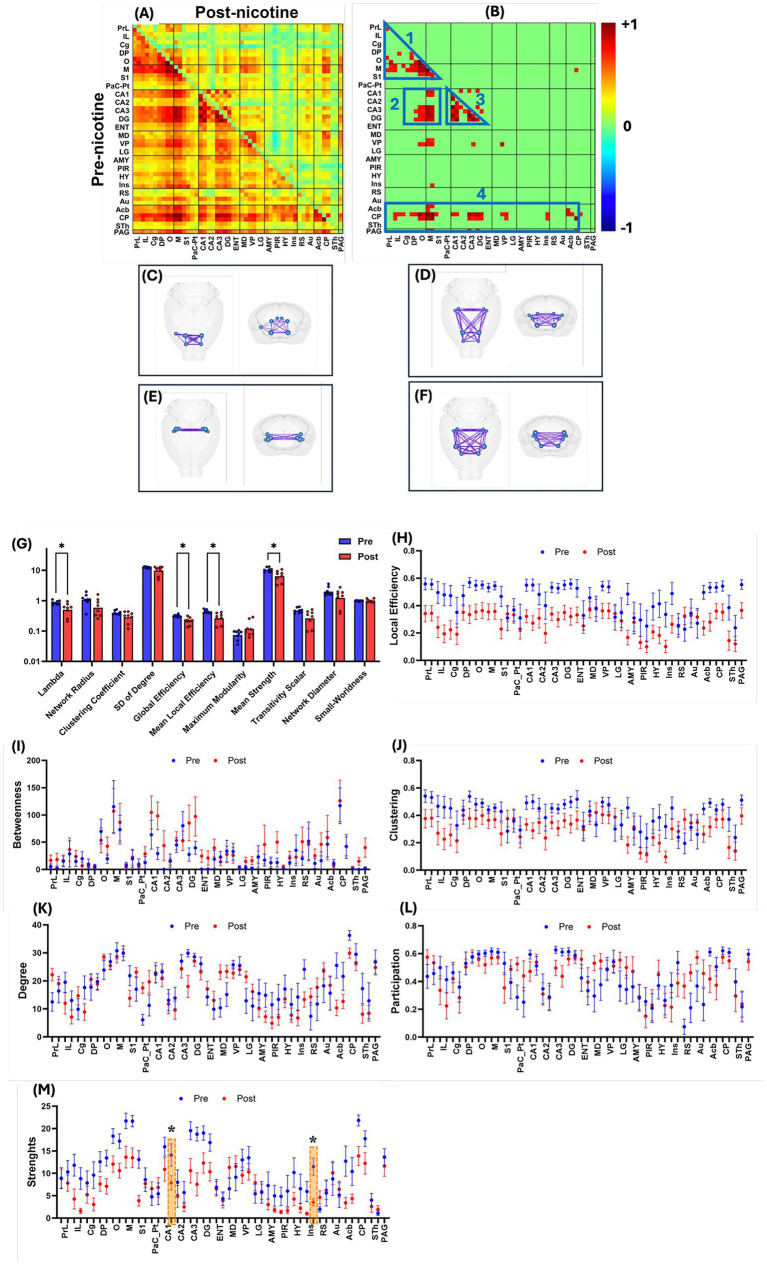
Glutamate-associated network organization before and after nicotine exposure (no GSR processing). **(A)** Group-averaged Fisher Z-transformed connectivity matrix derived from dynamic glutamate-weighted CEST time series without global signal regression (GSR). The lower-left triangle shows pre-nicotine connectivity, and the upper-right triangle shows post-nicotine connectivity. Strong cortico-cortical and cortico-hippocampal correlations present at baseline are visibly reduced after nicotine exposure. **(B)** Connectivity matrices thresholded to the top 5% of edges, revealing four dominant pre-nicotine modules: Module 1 (frontal–sensorimotor cortex), Module 2 (cortico-hippocampal network), Module 3 (intra-hippocampal connectivity), and Module 4 (striatal–midbrain network). All four modules are diminished post-nicotine, indicating widespread attenuation of glutamate-associated coupling. **(C–F)** Three-dimensional node–edge renderings of Modules 1–4. Node size represents regional involvement and edge thickness corresponds to Fisher Z-score, illustrating reduced within- and between-module connectivity following nicotine. (**G**) Global graph theory metrics comparing pre- and post-nicotine networks. Significant nicotine-related reductions were observed in Characteristic Path Length (Lambda), Global Efficiency, Local Efficiency, and Mean Strength (FDR-corrected *p* < 0.05). (**H–M**) Regional node-wise metrics across 51 brain regions: (**H**) Local Efficiency, **(I)** Betweenness Centrality, **(J)** Clustering Coefficient, **(K)** Degree, (**L**) Participation Coefficient, and **(M)** Nodal Strength. Nicotine selectively reduced Strength in right CA1 and right insular cortex (Ins) (FDR-corrected *p* < 0.05), reflecting localized vulnerability within hippocampal and insular nodes. Error bars represent SEM.

Figure 2A presents Fisher Z-transformed glutamate-associated connectivity matrices comparing pre- and post-nicotine conditions using glutamate-weighted time series without GSR. The lower-left triangle represents the group-averaged pre-nicotine connectivity matrix, while the upper-right triangle depicts the post-nicotine matrix. Regions were ordered along both axes according to their anatomical proximity to facilitate interpretation of network structure. Each anatomical label corresponds to two adjacent rows/columns representing the left and right hemispheric ROIs, respectively, except for the periaqueductal gray (PAG), which is a midline structure represented by a single row/column. Each matrix element represents the correlation strength between two regions, with diagonal elements (self-connections) set to zero. Nicotine caused a substantial reduction in multiple strong cortico-cortical and cortico-hippocampal connections evident under baseline conditions, as indicated by fewer high-strength connections in the upper-right triangle relative to the lower-left.

To highlight the most prominent network structures, matrices were thresholded to retain only the strongest 5% of connections (Figure 2B). In the pre-nicotine condition, four major connectivity modules were identified. Module 1 consisted of frontal and sensorimotor cortices, including the dorsal peduncular cortex (DP), orbital cortex (ORB), motor cortex (M), infralimbic cortex (IL), cingulate cortex (Cg), and primary somatosensory cortex (S1). Module 2 reflected cortico-hippocampal connectivity, linking the motor (M) and orbital (O) cortices with hippocampal subregions (CA1, CA2, CA3, and dentate gyrus (DG)). Module 3 represented the connectivity within the hippocampal region. Module 4 encompassed cortical, hippocampal, and striatal regions, forming an integrated cortico-hippocampal-striatal network. These network modules are illustrated using node-edge plots in Figures 2C–F, respectively, in which node size is proportional to ROI size (brain structure volume), and edge thickness is proportional to Fisher Z-transformed connectivity strength. Following nicotine administration, these pre-existing networks were markedly attenuated, indicating nicotine-induced decoupling of cortical and hippocampal structures.

Paired t tests with FDR correction were performed to compare global connectivity metrics, including Characteristic Path Length (Lambda), Network Radius, Clustering Coefficient, Standard Deviation (SD) of Degree, Global Efficiency, Mean Local Efficiency, Maximum Modularity, Mean Strength, Transitivity Scalar, Network Diameter, and Small-Worldness. Definitions of the metrics are provided in Supplementary Table 2. Significant nicotine-induced changes were observed in several global network measures, including Characteristic Path Length (Lambda) (corrected *p* = 0.042), Global Efficiency (corrected *p* = 0.033), Local Efficiency (corrected *p* = 0.036), and Mean Strength (corrected *p* = 0.047). The reduction in Global Efficiency, Local Efficiency, and Mean Strength indicate weakened network integration and reduced overall connectivity strength. In contrast, the decrease in Characteristic Path Length (Lambda) is interpreted in the context of sparse proportional thresholding as reflecting altered network topology and increased stochasticity or partial randomization of the retained network structure following nicotine exposure, rather than reduced communication efficiency alone. Local metrics including Local Efficiency, Betweenness, Clustering, Degree, Participation and Strength were compared for each node (brain region) (Figures 2H–M). Significant reduction was found in Strength on right CA1 (corrected *p* = 0.032) and right insular cortex (Ins, corrected *p* = 0.004) indicating weakened connectivity on these regions following nicotine exposure.

#### 3.2.2 Data conditionally processed with GSR

**Figure 3:**
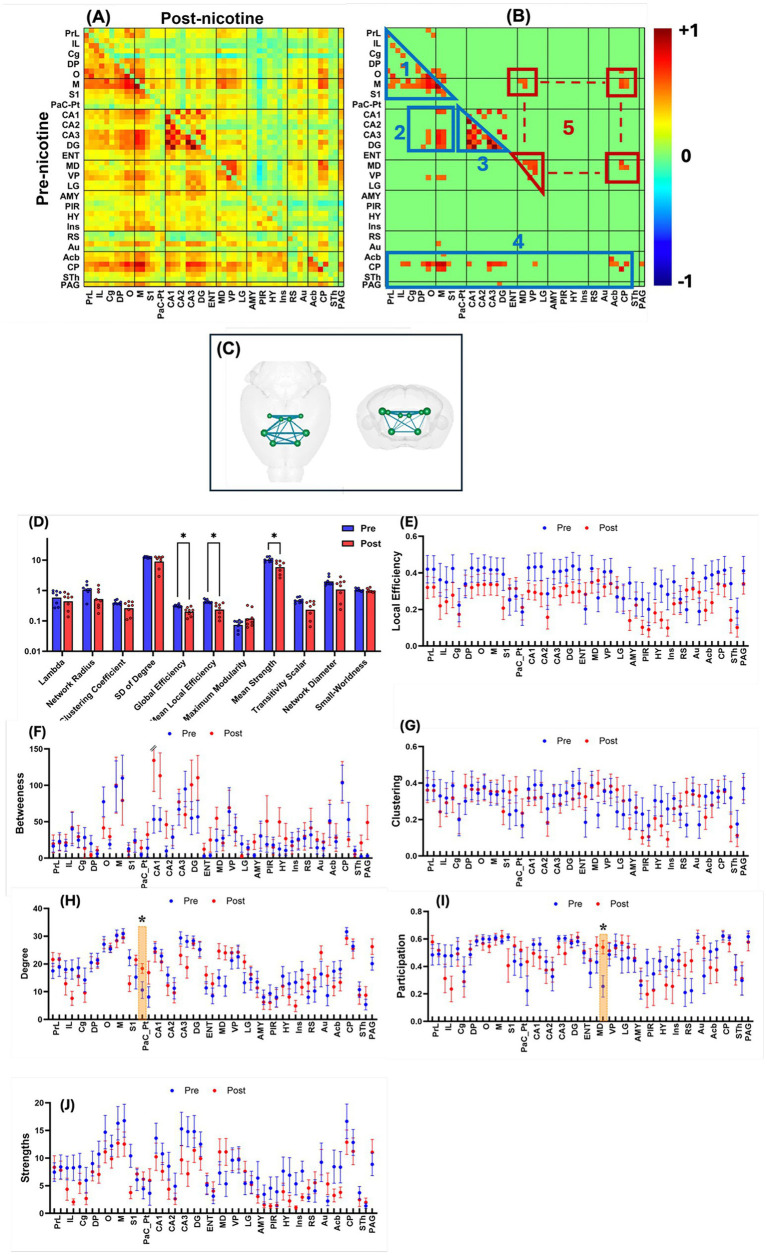
Glutamate-associated network organization before and after nicotine exposure (conditional GSR processing). **(A)** Group-averaged Fisher Z-transformed connectivity matrices generated from dynamic glutamate-weighted CEST time series processed with global signal regression (GSR) applied only when the global negative index (GNI) < 3. The lower-left triangle shows pre-nicotine connectivity and the upper-right triangle shows post-nicotine connectivity. Compared with the no-GSR analysis, conditional GSR reduces several baseline correlations but preserves the major network structures, while post-nicotine patterns remain largely unchanged. **(B)** Connectivity matrices thresholded to the top 5% of edges, highlighting dominant pre- and post-nicotine modules. Four pre-nicotine modules (1–4) largely match those identified in the no-GSR analysis. Nicotine attenuates these cortico-hippocampal and intra-hippocampal modules and selectively enhances a distinct thalamo–striato–motor circuit (Module 5, dashed box), comprising motor cortex (M), mediodorsal (MD) and ventral posterior (VP) thalamic nuclei, and caudoputamen (CPu). **(C)** Three-dimensional node–edge rendering of Module 5, illustrating the nicotine-enhanced thalamo–striato–motor network. Node size reflects regional involvement, and edge thickness reflects connection strength proportional to the Fisher Z-score. **(D)** Global graph theory metrics comparing pre- and post-nicotine networks. Although many metrics show minimal group-level differences, nicotine induces significant reductions in Global Efficiency, Local Efficiency, and Strength (indicated by asterisks). **(E–J)** Regional node-wise metrics for each of the 51 brain regions: **(E)** Local Efficiency, **(F)** Betweenness, **(G)** Clustering Coefficient, **(H)** Degree, **(I)** Participation Coefficient, and **(J)** Strength. Most nodal metrics remain unchanged following nicotine exposure, except for a significant increase in Parietal Cortex (Pac/Pt) on Degree, and in the mediodorsal thalamus (MD) on Participation Coefficient. Error bars represent SEM.

Glutamate-associated connectivity matrices calculated using glutamate-weighted time series *with GSR when GNI < 3* are shown in Figure 3A. The GNI values for all datasets are provided in Supplementary Table 1. Visual inspection indicated that, under the baseline (pre-nicotine) condition, GSR attenuated several correlations, resulting in an overall reduction in connectivity strength. In contrast, the post-nicotine connectivity matrix was largely unchanged by GSR (Figure 3A), suggesting that nicotine-induced network alterations were robust to global signal removal. The top 5% of Fisher Z-scores is shown in Figure 3B. In the pre-nicotine condition, four distinct connectivity modules (Module 1–4 in Figure 3B) emerged, closely matching the modular structure identified in the data processed without GSR shown in Figure 2B. Consistent with the findings from analyses without GSR, these modules were diminished following nicotine administration, reflecting nicotine-induced disruption of glutamate-associated connectivity. Conversely, nicotine enhanced connectivity in a distinct set of regions involving the motor cortex (M), thalamus (mediodorsal nucleus [MD] and ventral posterior nucleus [VP]), and caudatoputamen (CPu), forming a thalamo-striato-motor axis (Module 5 in Figure 3B). These findings suggested that nicotine suppressed baseline cortico-hippocampal connectivity while selectively enhancing thalamo-striatal and motor-related pathways, highlighting a reorganization of glutamate-associated network topology. Module 5 is illustrated using node-edge plots in Figure 3C, Node size is proportional to ROI size (brain structure volume), and edge thickness is proportional to Fisher Z-transformed connectivity strength.

Three global connectivity metrics exhibited significant nicotine-related reductions (Figure 3D): Global Efficiency (corrected *p* = 0.029), Local Efficiency (corrected *p* = 0.031), and Mean Strength (corrected *p* = 0.042). These decreases indicated diminished integration and weakened connectivity strength following nicotine exposure. Statistical analysis on nodal connectivity metrics (Figures 3E–J) showed that nicotine exposure significantly increased node Degree in the left Parietal Cortex (PaC/Pt, corrected *p* = 0.027, Figure 3H), indicating enhanced local connectivity of this region after nicotine administration. Additionally, the Participation Coefficient, which reflects how broadly a node distributes its connections across different network modules, was significantly elevated in the right mediodorsal thalamus (MD, corrected *p* = 0.019, Figure 3I), suggesting enhanced cross-modular communication of this thalamic hub following nicotine exposure. These statistical results were consistent with the correlation matrices in Figures 3A,B. Nicotine attenuated global connectivity, reducing overall communication efficiency and connection strength, while selectively enhanced certain nodal metrics, such as Degree and Participation Coefficient, indicating strengthened cortico-thalamic communication.

#### 3.2.3 Data processed with GSR

**Figure 4:**
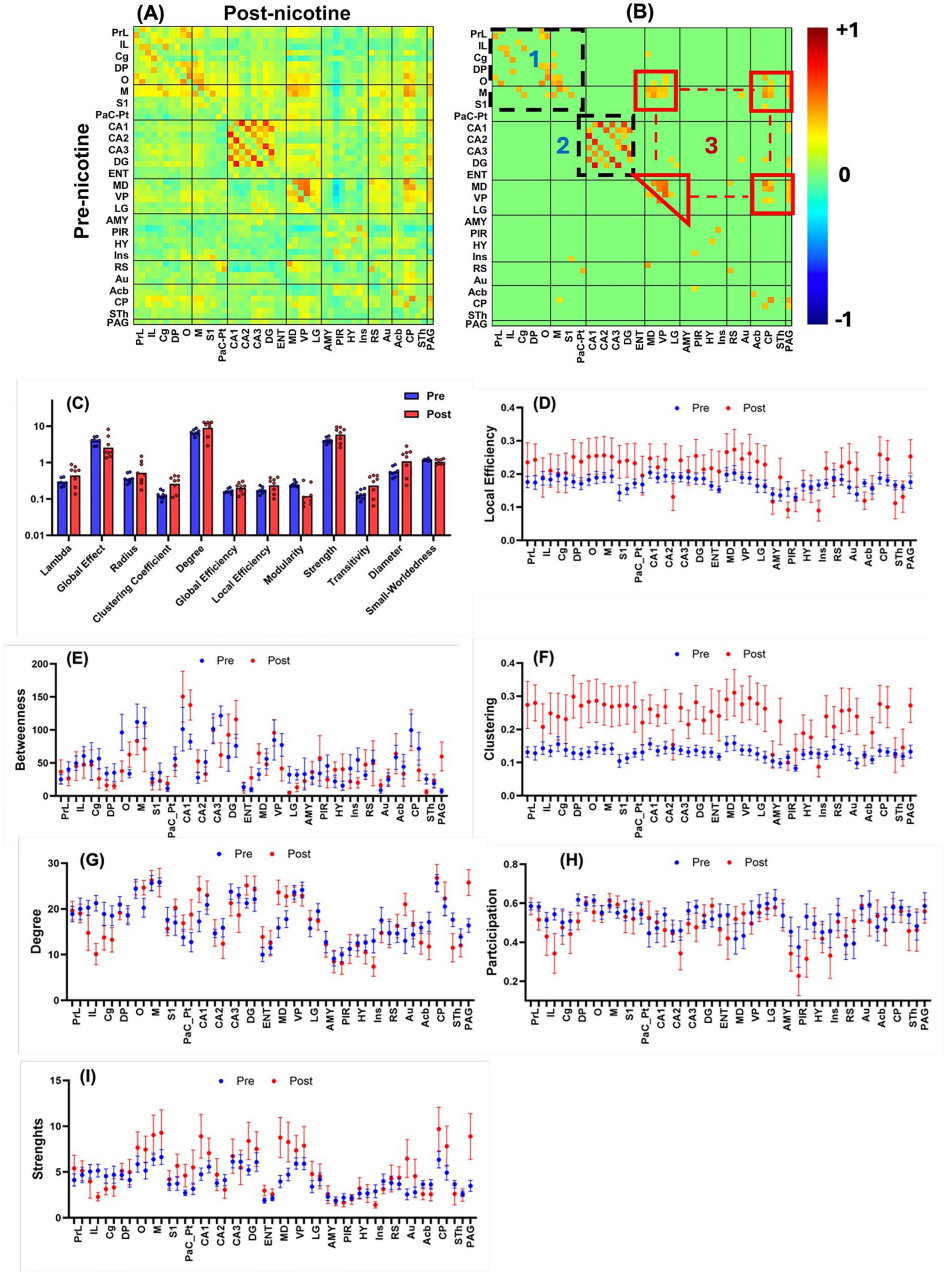
Glutamate-associated network organization before and after nicotine exposure (full GSR processing). **(A)** Group-averaged Fisher Z-transformed connectivity matrices generated from dynamic glutamate-weighted CEST time series processed with full global signal regression (GSR). The lower-left triangle represents pre-nicotine connectivity, while the upper-right triangle shows post-nicotine connectivity. Full GSR further attenuates correlation strengths relative to both the no-GSR and conditional-GSR analyses, with reduced contrast across many brain regions. **(B)** Connectivity matrices thresholded to the top 5% of edges, highlighting the dominant network modules. Two major pre-nicotine modules (Modules 1 and 2) remain clearly identifiable and correspond to those observed in Figures 2, 3. A nicotine-enhanced thalamo–striato–motor circuit (Module 3), involving motor cortex (M), mediodorsal (MD) and ventral posterior (VP) thalamic nuclei, and caudoputamen (CPu), is also evident. All module structures shown here were previously illustrated in Figures 2, 3; therefore, node–edge renderings are not repeated. **(C)** Global graph theory metrics comparing pre- and post-nicotine networks. Under full GSR, none of the global measures including global/local efficiency, clustering coefficient, degree, modularity, transitivity, diameter, radius, and small-worldness, show statistically significant differences between conditions. **(D–I)** Regional node-wise metrics for each of the 51 brain regions: **(D)** local efficiency, **(E)** betweenness centrality, **(F)** clustering coefficient, **(G)** degree, **(H)** participation coefficient, and **(I)** nodal strength. Across nodes, pre- and post-nicotine values remain largely similar, with no significant regional differences detected following FDR correction. Error bars represent SEM.

Figure 4A shows glutamate-associated connectivity matrices calculated using glutamate-associated time series *with* GSR. Compared to the conditionally applied GSR, both pre- and post-nicotine correlations were further attenuated by full GSR. In the top 5% of matrix elements (Figure 4B), Modules 1 and 2 were still identifiable in the pre-nicotine condition, consistent with the modules observed in the analyses without GSR and with conditional GSR (Figures 2B, 3B). The two modules remained evident after nicotine exposure and did not show the marked attenuation observed in the non-GSR and conditional-GSR analyses (Figures 2B, 3B). Similarly to the results analyzed with conditional GSR, Module 3 showed enhanced connectivity among motor cortex (M), thalamus (mediodorsal nucleus [MD] and ventral posterior nucleus [VP]), and caudatoputamen (CPu). No significant difference was found on global and local metrics between pre- and post-nicotine groups (Figures 4C–I). Unlike the analyses without GSR and with conditional GSR, full GSR reduced the apparent magnitude of nicotine-induced attenuation in baseline cortico-hippocampal modules, suggesting that some nicotine-sensitive glutamate-associated fluctuations may reside within widespread global signal components.

### 3.3 Subject-level (inter-subject) connectivity analysis

**Figure 5:**
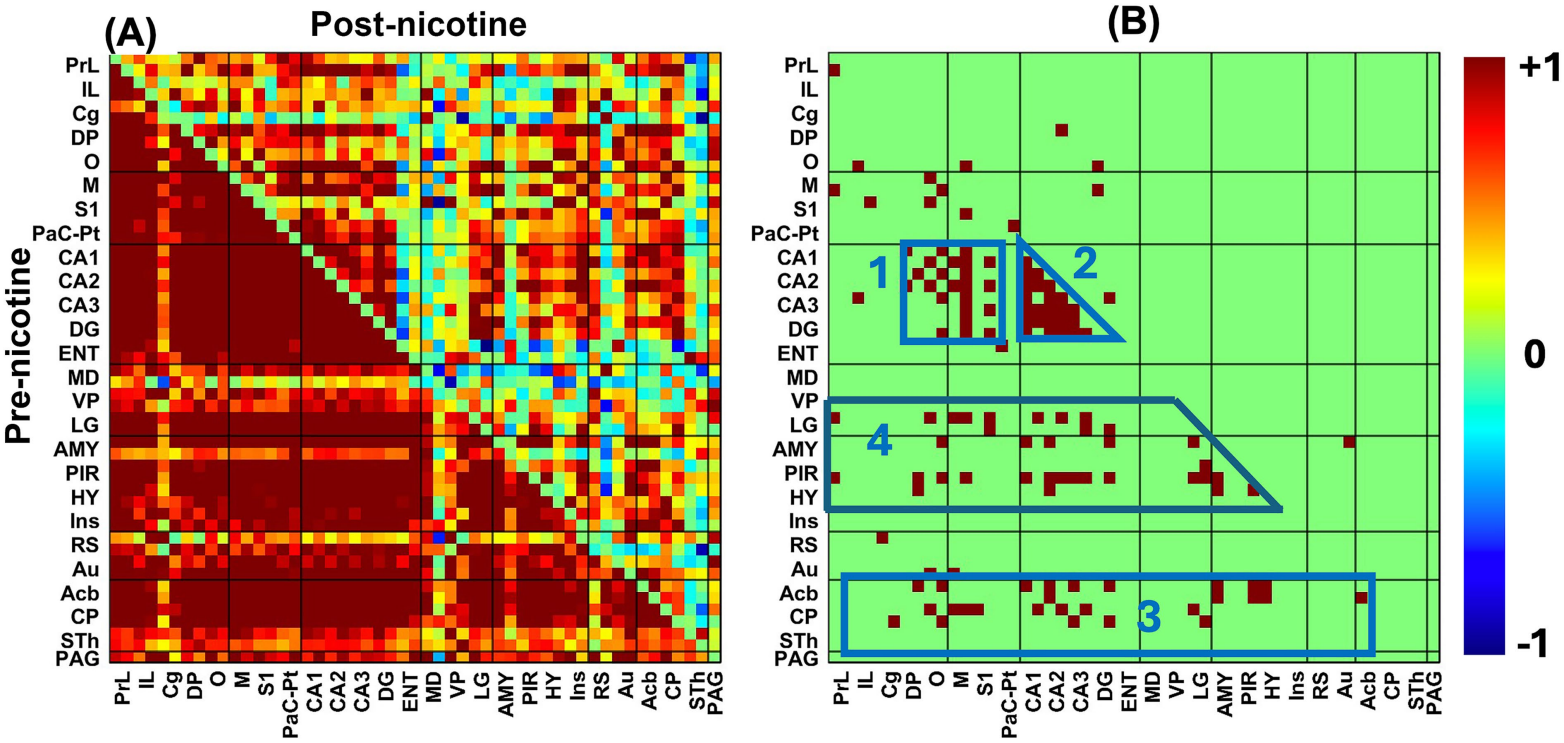
Subject-level (inter-subject) glutamate-associated connectivity before and after nicotine exposure. **(A)** Subject-level Fisher Z-transformed connectivity matrices computed by correlating regional mean gluCEST values across animals (one value per region per subject). The lower-left triangle shows pre-nicotine connectivity, and the upper-right triangle shows post-nicotine connectivity. Before nicotine exposure, strong positive correlations are evident across nearly all regions. Nicotine exposure attenuates many of these correlations. **(B)** Connectivity matrices thresholded to the top 5% of edges, revealing four dominant pre-nicotine modules. Modules 1, 2, and 3 closely match the network structures identified in time-series analyses without GSR and with conditional GSR (Figures 2, 3), confirming the robustness of these glutamate-associated circuits across analytic approaches. Module 4, consisting of strong connectivity among lateral geniculate nucleus (LG), amygdala (AMY), and piriform cortex (PIR), is unique to the subject-level covariance analysis. All four modules are markedly suppressed after nicotine administration.

Before nicotine exposure, subject-level correlations revealed strong positive relationships across nearly all brain regions, indicating highly covarying glutamate contrast patterns between animals (Figure 5A). Following nicotine administration, several of these inter-regional correlations were attenuated, suggesting reduced glutamate activity. When restricting the analysis to the top 5% of matrix elements (Figure 5B), four distinct modules were identified in the pre-nicotine condition. Three of these modules (Modules 1, 2, and 3) corresponded closely to those observed in the time-series analyses without GSR and with conditional GSR (Figures 2, 3), demonstrating that similar network structures emerged across analytical approaches. A fourth module, unique to the subject-level correlation analysis, consisted of strong connectivity among the lateral geniculate nucleus (LG) and limbic-associated regions including the amygdala (AMY) and piriform cortex (PIR). All these modules were markedly suppressed following nicotine exposure, indicating nicotine-induced disruption of coordinated glutamate activity across subjects in these circuits.

## 4 Discussion

Here we presented a novel CEST MRI framework for mapping brain molecular connectivity using glutamate-weighted signals and applied it to reveal nicotine-induced reorganization of glutamate-associated networks. Across multiple analytic strategies, including within-subject temporal correlations, conditional versus full GSR, and subject-level covariance analyses, the data demonstrated robust nicotine-associated network alterations in analyses without GSR and with conditional GSR, whereas several effects were attenuated under full GSR. These findings suggest that CEST-derived molecular networks provide metabolite-weighted information complementary to conventional fMRI and structural connectivity approaches.

### 4.1 Nicotine selectively disrupts cortico-hippocampal glutamate-associated connectivity

Across analyses without GSR and with conditional GSR, the strongest baseline glutamate-associated connections involved frontal, motor, somatosensory, and hippocampal structures (Modules 1–4 in Figures 2B, 3B). These networks resemble well-defined excitatory pathways implicated in cognitive control, sensorimotor integration, and memory processing. Nicotine consistently attenuated these pre-existing networks, reflected by reductions in high strength edges and diminished modular structure. This effect was most striking in analyses without GSR and with conditional GSR, where multiple cortico-hippocampal modules nearly disappeared post-nicotine (Figures 2B, 3B). The reproducibility of this effect across processing pipelines strengthens the conclusion that nicotine reduces coordinated glutamate fluctuations among these regions. Local reductions in nodal strength, particularly in CA1 and insular cortex, further support nicotine-induced disruptions in intrahippocampal and interoceptive networks, which integrate internal physiological signals and contribute to autonomic regulation, emotional state, and craving-related processes. These findings align with known nicotine-induced modulation of glutamatergic synaptic transmission and hippocampal plasticity (Ji et al., 2001; McGehee, 2002), and they highlight the sensitivity of CEST MRI to neurochemical network reorganization.

### 4.2 Nicotine enhances thalamo-striato–motor glutamate-associated connectivity

In contrast to the widespread decreases noted above, nicotine reliably strengthened a distinct set of connections linking the motor cortex, mediodorsal and ventral posterior thalamic nuclei, and caudoputamen (Module 5 in Figures 3B and Module 3 in Figure 4B). This pattern resembles a thalamo-striato-motor axis implicated in motor preparation, sensorimotor gating, and action selection. The enhancement of these circuits by nicotine is consistent with prior electrophysiological and neuroimaging work showing nicotine-induced activation of thalamic and cortico-basal ganglia-thalamic loops, as well as nicotine-evoked increases in striatal glutamate release and dopamine-glutamate interactions in nigrostriatal and mesolimbic pathways (Perl et al., 2024; Kim et al., 2019; Stein et al., 1998; Lawrence et al., 2002; Sharma and Brody, 2009; Garcia-Munoz et al., 1996; Gao et al., 2010). Of note, this nicotine-enhanced module was preserved even when full GSR was applied, suggesting that these circuits exhibit robust glutamate-weighted signal coupling that is not dependent on global fluctuations. This contrasted with the fragility of cortico-hippocampal modules, which were highly sensitive to both GSR and nicotine exposure.

### 4.3 Global and nodal metrics reveal nicotine-induced network reorganization alongside pathway-specific effects

Global graph theory measures revealed clear and biologically meaningful nicotine-induced alterations in network organization. In the dataset without GSR, several whole-network metrics, including Characteristic Path Length (Lambda), Global Efficiency, Local Efficiency, and Mean Strength, showed significant changes after nicotine exposure (Figure 2G). Although reductions in Global Efficiency, Local Efficiency, and Mean Strength suggest weakened network integration, the decrease in Characteristic Path Length (Lambda) should not be interpreted independently as reduced communication efficiency. Under sparse proportional thresholding, reduced Lambda may instead reflect increased stochasticity or partial randomization of retained network topology following nicotine exposure. These effects were reproduced under conditional GSR (Figure 3D), where Global Efficiency, Local Efficiency, and Mean Strength decreased significantly, demonstrating that nicotine disrupts core organizational properties of glutamate-associated networks even when global fluctuations are partly removed. Only full GSR eliminated these differences (Figure 4), indicating sensitivity of the observed network effects to preprocessing strategy, particularly the handling of global signal components. At the same time, nodal analyses revealed that these global alterations manifested as consistent weakening of cortico-hippocampal pathways (Figure 2M) alongside selective strengthening of a thalamo-striato-motor circuit (Figures 3H, I). Together, these results show that nicotine alters both global topological properties and specific brain regions.

### 4.4 Subject-level covariance analysis reveals cross-animal consistency in glutamate-associated network alterations

Subject-level (inter-subject) connectivity matrices exhibited a different perspective: strong global covariance across animals pre-nicotine, followed by widespread attenuation after nicotine (Figure 5A). Importantly, three of the four modules identified in subject-level analysis aligned with corresponding modules in temporal analyses (Figure 5B vs. Figures 2–4), demonstrating that certain glutamate-associated networks are robust across both intra- and inter-individual variability. Module 4 in the subject-level matrices, linking the lateral geniculate nucleus (LG), amygdala (AMY), and piriform cortex (PIR), was unique to this analysis and disappeared post-nicotine. This suggests coordinated sensory-limbic glutamate alterations among animals that are disrupted following nicotine exposure. The LG participates in sensory relay and salience-related processing, whereas the amygdala and piriform cortex are strongly implicated in emotional regulation, associative learning, and olfactory-limbic circuitry. Unlike the more prominent cortico-hippocampal and thalamo-striatal modules identified in the temporal analyses, this LG-AMY-PIR network may represent a distinct sensory-limbic component of nicotine-associated network remodeling, potentially related to salience and affective processing. However, the functional interpretation of this module remains preliminary given the exploratory nature of the current study and limited sample size, and future studies incorporating behavioral paradigms and longitudinal molecular connectivity analysis will be important to further define its biological significance.

### 4.5 CEST MRI as a tool for molecular network mapping

The consistency of nicotine-induced changes across multiple analyses underscores the promise of CEST MRI for probing molecular networks. Unlike BOLD fMRI, which reflects hemodynamic responses, CEST directly captures metabolite-weighted contrast at high spatial resolution and without exogenous tracers. Compared to PET, CEST avoids radiotracers, improves spatial specificity, and enables repeated scanning within a short timeframe. By mapping molecular connectivity at the level of individual subjects, this approach provides a complementary link between metabolic, neurotransmitter, and functional imaging. The demonstrated sensitivity to both attenuated and enhanced nicotine-induced network changes suggests that CEST MRI may be a valuable tool for studying pharmacological modulation, addiction circuits, neurometabolic disorders, and therapeutic interventions.

### 4.6 Effects of GSR

Our use of both GSR-free and GSR-based processing pipelines acknowledges the complex and still debated role of the global signal in functional neuroimaging. The global signal, defined as the brain wide average signal, reflects a mixture of neural, vascular, and physiological fluctuations, and its removal via GSR can have substantial effects on correlation-based connectivity estimates. Multiple studies have shown that GSR reduces non-neural noise (e.g., motion, respiration, scanner drift), thereby improving spatial specificity and strengthening associations between connectivity patterns and behavior, cognition, or clinical traits (Murphy and Fox, 2017; Li et al., 2019; Liu et al., 2017). At the same time, GSR is mathematically guaranteed to re-center correlation distributions around zero and frequently introduces negative correlations (anti-correlations), raising major concerns about the biological interpretability of such effects (Saad et al., 2012; Murphy et al., 2009). Beyond technical artifacts, emerging evidence suggests that the global signal contains genuine neural and vascular contributions. For instance, studies indicate that global brain activity including slow fluctuations in cortical and systemic physiology correlates with arousal, autonomic state, and even bone/vascular signal components, implying that some of what is removed by GSR may be biologically meaningful (Liu et al., 2017; Huber et al., 2024; Ao et al., 2021). Given this trade-off, GSR may simultaneously reduce noise and eliminate physiological or neural information. Additional nuisance regressors, such as white matter or ventricular signals, may further refine separation of global and region-specific fluctuations. However, these approaches are not yet routinely used in small animal imaging because limited tissue contrast and the small size of ventricular and white matter structures reduce segmentation reliability. Future work incorporating improved anatomical segmentation and tissue-specific nuisance regression may further improve characterization of glutamate-associated connectivity.

Applying this to our glutamate-associated network analyses, the decision to present results both with and without GSR enables more nuanced interpretation. Changes that persist across both pipelines (e.g., nicotine-induced weakening of cortico-hippocampal connectivity, strengthening of thalamo-striatal networks) are more likely to reflect true neurometabolic coupling rather than preprocessing artifacts. Conversely, effects that emerge only after GSR should be interpreted cautiously, because GSR may have artificially altered the correlation structure. Ultimately, the use (or avoidance) of GSR does not simply improve or degrade data. It changes what aspect of brain function we choose to emphasize. In CEST-based molecular connectivity mapping, this bifurcated approach may help disentangle widespread non-specific fluctuations from regionally structured glutamate-weighted signaling.

The attenuation of several nicotine-induced effects under full GSR suggests that widespread glutamate-associated fluctuations may themselves contain biologically meaningful information rather than representing purely non-neural noise. Accordingly, full GSR may remove not only global noise but also biologically relevant widespread glutamate-associated fluctuations, thereby reducing sensitivity to nicotine-induced network changes.

### 4.7 Limitations

Several limitations warrant consideration. First, connectivity derived from dynamic gluCEST reflects coordinated fluctuations in glutamate-associated contrast rather than direct neuronal coupling; therefore, mechanistic interpretation requires complementary electrophysiological or molecular validation. In addition, gluCEST reflects glutamate-associated tissue contrast rather than a direct measure of synaptic glutamate release. The signal likely represents a combination of intracellular and extracellular glutamate pools, with intracellular glutamate expected to dominate because of its substantially higher concentration in brain tissue.

Second, although glutamate is the predominant contributor to the 3.0 ppm CEST effect, minor contributions from macromolecular resonances cannot be completely excluded. Network results may also depend on preprocessing choices such as GSR and thresholding; however, the convergence of findings across multiple preprocessing pipelines in this study strengthens the reliability of the major observations.

Third, seed-based validation was performed using a single reference region (right S1bf) as suggested by recent multi-center consensus analyses (Grandjean et al., 2023). Although this approach provided an intuitive quality assessment of anatomically plausible connectivity, it does not comprehensively evaluate all network structures and should be interpreted as an initial validation step.

Fourth, although dynamic gluCEST signals could theoretically be influenced by hemodynamic (BOLD-related) effects, several features of the acquisition reduce sensitivity to canonical BOLD fluctuations. The CEST contrast at 3.0 ppm is frequency-specific and generated through chemical exchange rather than susceptibility changes. In addition, the long RF saturation pulses, off-resonance irradiation, spin-echo readout, and relatively slow temporal resolution (35 s) all reduce sensitivity to T2*-driven BOLD fluctuations. Nevertheless, residual hemodynamic contributions cannot be completely excluded. In addition, slow signal variation related to B0 drift, anesthesia depth, or physiological fluctuation may contribute to the dynamic gluCEST time series despite interpolation-based frequency correction and physiologic monitoring.

Anesthesia is another important consideration when interpreting dynamic gluCEST connectivity. Prior rodent resting-state fMRI (rs-fMRI) studies have shown that anesthetic conditions and physiologic monitoring can substantially influence connectivity measurements and their reproducibility (Belloy et al., 2018; Grandjean et al., 2020; Gutierrez-Barragan et al., 2019; Özalay et al., 2024). In this study, imaging was performed under isoflurane anesthesia with continuous monitoring of respiratory rate and body temperature, and the same anesthesia preparation and monitoring procedures were applied across baseline and post-nicotine scans to minimize inter-session variability. Because dynamic gluCEST measures glutamate-associated molecular contrast rather than hemodynamic BOLD fluctuations, the physiologic sensitivity and anesthetic requirements may differ from conventional rs-fMRI. No substantial temporal drift was visually observed across the dynamic gluCEST time series, and detrending was therefore not applied. However, formal temporal segmentation of the dynamic datasets to further evaluate potential anesthesia-related drift during scanning was not performed in the present study and would be valuable in future work. In addition, subtle interactions among anesthesia, physiologic state, and nicotine exposure cannot be completely excluded and may contribute to the observed connectivity patterns. Future studies incorporating more detailed physiologic measurements, temporal stability analyses, and complementary imaging modalities will be important to further distinguish glutamate-associated network effects from anesthesia-related influences.

Additional limitations include the relatively small sample size (*n* = 8) and the use of only male mice, which may limit statistical power and generalizability. Some nodal-level findings may therefore be sensitive to inter-subject variability and should be validated in larger, sex-balanced cohorts. In addition, because this study was performed in a single inbred strain (C57BL/6), strain-specific neurobiological characteristics may influence glutamate-associated connectivity patterns and should be considered in future validation studies across additional animal models.

Finally, nicotine pharmacokinetics were not directly evaluated in this study. Imaging was performed one hour after the final nicotine injection to reduce acute injection-related physiological effects while emphasizing downstream neurometabolic network alterations associated with repeated nicotine exposure rather than direct measurement of acute nicotine concentration. In addition, dedicated behavioral testing was not performed in this study. Therefore, the observed imaging findings could not be directly correlated with nicotine-related behavioral phenotypes or sedation effects, and future studies incorporating behavioral assessments will be important for translational interpretation.

### 4.8 Future directions

In future work, we aim to map multi-metabolite CEST networks (including creatine, glutamate, glucose, and myo-inositol) from a single multi-offset CEST acquisition. This will be enabled by AI-accelerated acquisition strategies, in which data are collected at optimally selected sparse frequency offsets and complete voxel-wise Z-spectra are reconstructed using deep learning methods (Bhattarai et al., 2025; Bhattarai et al., 2025). These approaches allow dynamic CEST imaging with temporal resolution comparable to the single-frequency method used in the present study, while providing metabolite-associated network information across the full frequency spectrum.

The framework demonstrated here in mice has strong potential for translation to human imaging, particularly on high field 7 T MRI systems where CEST sensitivity and spectral dispersion are substantially improved. Human 7 T studies have already established the feasibility of glutamate-weighted CEST imaging (Debnath et al., 2020; Hadar et al., 2021), and dynamic acquisitions similar to those used here could enable subject-specific molecular connectivity mapping in clinical populations. Important considerations for translation include B0/B1 inhomogeneity correction, motion mitigation, and optimization of temporal resolution; however, none pose conceptual barriers. Ultimately, human molecular connectivity mapping may provide new biomarkers for disorders characterized by altered glutamatergic signaling, including addiction, schizophrenia, depression, and neurodegeneration.

This molecular connectivity approach also provides opportunities for multimodal integration. PET imaging of glucose metabolism or neurotransmitter receptor binding could help validate CEST-derived networks and clarify whether nicotine-induced changes reflect altered neurotransmission, metabolism, or both. Electrophysiological recordings or calcium imaging could link CEST connectivity to underlying neuronal activity and synaptic dynamics. Simultaneous or parallel fMRI acquisitions may help further define the relationship between metabolite-weighted and hemodynamic connectivity signals. Combining CEST MRI with these modalities would offer a multidimensional view of glutamatergic function, allowing mechanistic interpretation of network alterations and facilitating cross-validation of emerging molecular connectomics.

## 5 Conclusion

This study demonstrates the feasibility of glutamate-weighted CEST MRI for mapping glutamate-associated molecular connectivity *in vivo*. The ability to noninvasively monitor molecular circuits at high spatial resolution opens new avenues for studying brain organization in health, disease, and therapeutic response. These findings support dynamic gluCEST MRI as a promising platform for investigating glutamate-associated network alterations in preclinical models and for future translational studies.

## Data Availability

The original contributions presented in the study are included in the article/Supplementary material, further inquiries can be directed to the corresponding author.

## Glossary

PrL: Prelimbic cortex
IL: Infralimbic cortex
Cg: Cingulate cortex
DP: Dorsal peduncular cortex
O: Orbital cortex
M: Motor cortex
S1: Primary somatosensory cortex
PaC/Pt: Parietal Cortex
CA1: CA1 of hippocampus
CA2: CA2 of hippocampus
CA3: CA3 of hippocampus
DG: Dentate gyrus
ENT: Entorhinal Cortex
MD: Mediodorsal thalamic nucleus
VP: Ventral posterior thalamic nucleus
LG: Lateral geniculate nucleus
AMY: Amygdala
PIR: Piriform cortex
HY: Hypothalamus
Ins: Insular cortex
RS: Retrosplenial cortex
Au: Auditory cortex
ACB: Nucleus accumbens
CPu: Caudoputamen
STh: Subthalamic nucleus
PAG: Periaqueductal gray
