# Supplementary material for: CEST MRI reveals nicotine-induced alterations in glutamate-associated molecular connectivity in the mouse brain
Source: Front Neurosci. 2026 Jun 16;20:1815092. doi: 10.3389/fnins.2026.1815092 (PMC13315015; doi:10.3389/fnins.2026.1815092)
Supplement: Supplementary file 1 [file Data_Sheet_1.DOCX]

| Mouse # | Pre-nicotine | Post-nicotine |
| --- | --- | --- |
| 01 | 4.5 | 2.5 |
| 02 | 6.0 | 2.4 |
| 03 | 4.0 | 2.9 |
| 04 | 2.2 | 3.5 |
| 05 | 2.8 | 2.8 |
| 06 | 2.5 | 2.8 |
| 07 | 3.1 | 1.8 |
| 08 | 4.0 | 6.8 |

**Supplemental Table 1.** GNI of each mouse. GNI < 3 are in red color

**Supplemental Table 2.** Definitions of global connectivity metrics

| Global Metrics | Definitions |
| --- | --- |
| Characteristic Path Length (Lambda) | Average shortest-path distance between all node pairs, reflecting global communication efficiency. |
| Network Radius | The minimum eccentricity across nodes, indicating the distance from the most central node to the network. |
| SD of Degree | The standard deviation of node degree values, capturing variability in how many connections nodes possess. |
| Global Efficiency | The average inverse shortest-path length, measuring how efficiently information is transferred across the whole network. |
| Mean Local Efficiency | Average communication efficiency within each node’s local neighborhood, reflecting local segregation and robustness. |
| Maximum Modularity | The highest modularity score detected, representing how strongly the network partitions into distinct modules. |
| Mean Strength | Average sum of weighted connections per node, representing overall connection magnitude in the network. |
| Transitivity (Scalar) | Ratio of closed triangles to connected triplets, quantifying global clustering in the network. |
| Network Diameter | The maximum shortest-path distance between any two nodes, representing the longest minimal communication route. |
| Small-Worldness | Normalized ratio of clustering and path length relative to random networks, indicating small-world architecture. |

**Supplemental Table 3.** Definitions of nodal connectivity metrics

| Nodal Metrics | Definitions |
| --- | --- |
| Local Efficiency | **Efficiency of information transfer within a node’s immediate neighborhood, reflecting local resilience and segregation.** |
| Betweenness Centrality | The proportion of shortest paths in the network that pass through a node, indicating its role as a bridge or control point for communication. |
| Clustering Coefficient | The fraction of a node’s neighbors that are also interconnected, quantifying the node’s local cohesiveness. |
| Degree | The number of direct connections a node has to other nodes, representing its immediate connectivity level. |
| Participation Coefficient | A measure of how evenly a node’s connections are distributed across different modules, indicating its role in cross-module integration. |
| Strength | The sum of the weights of all connections linked to a node, reflecting the overall magnitude of its connectivity. |


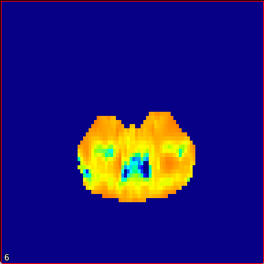

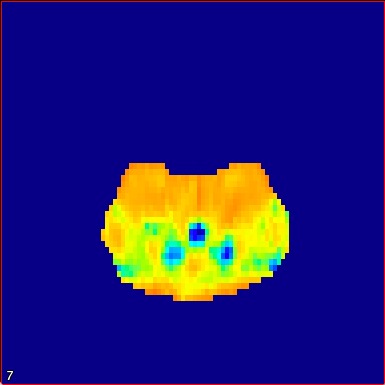

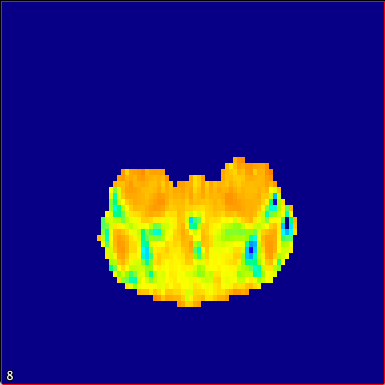

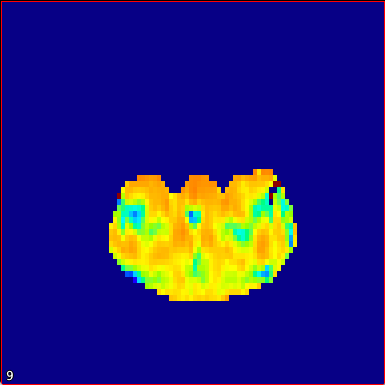

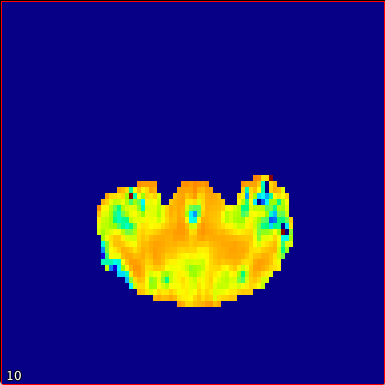


**(B) Post-nicotine glutamate-weighted contrast map**


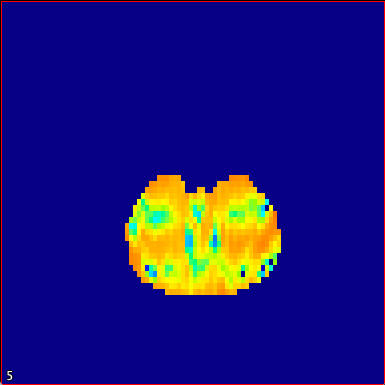

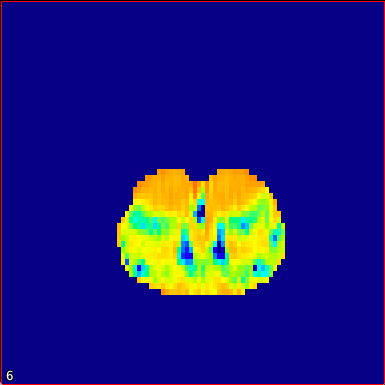

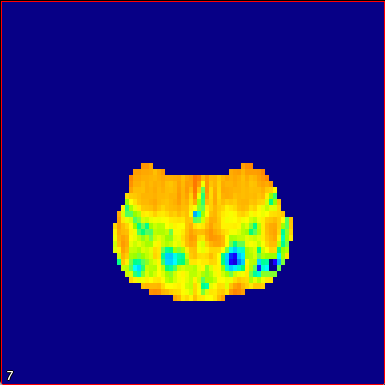

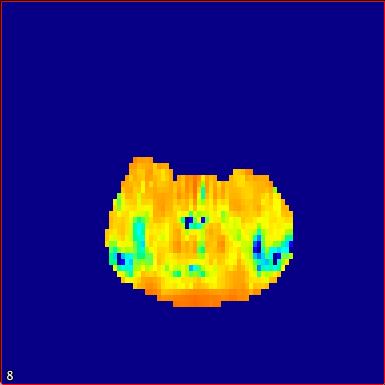

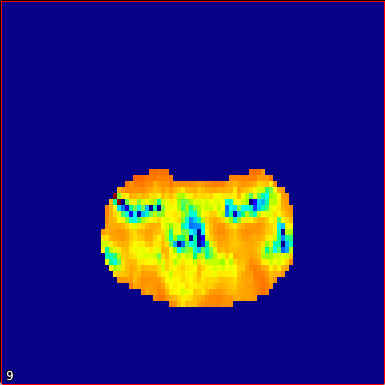


**(A) Pre-nicotine glutamate-weighted contrast map**


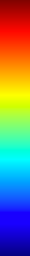


0.0

0.3

**Supplemental Figure 1.** Representative glutamate-weighted CEST (gluCEST) maps from a representative mouse acquired at baseline before nicotine administration (A) and one hour after the final nicotine administration (B), demonstrating the quality and spatial distribution of glutamate-associated contrast across the brain.
